# Effect of the Solid Solution and Aging Treatment on the Mechanical Properties and Microstructure of a Novel Al-Mg-Si Alloy

**DOI:** 10.3390/ma16217036

**Published:** 2023-11-04

**Authors:** Yan Chen, Wu Wei, Yu Zhao, Wei Shi, Xiaorong Zhou, Li Rong, Shengping Wen, Xiaolan Wu, Kunyuan Gao, Hui Huang, Zuoren Nie

**Affiliations:** 1 Key Laboratory of Advanced Functional Materials, Education Ministry of China, Beijing University of Technology, Beijing 100124, China; 2 Institute of Corrosion Science and Technology, Guangzhou 510530, China; 3 Department of Materials, The University of Manchester, Manchester M13 9PL, UK

**Keywords:** Al-Mg-Si alloy, solid solution and aging treatment, Mg_2_Si phases, Al_3_(Er,Zr) phases, precipitation behavior

## Abstract

A novel Al-Mg-Si aluminum alloy with the addition of the micro-alloying element Er and Zr that was promptly quenched after extrusion has been studied. The solid solution and aging treatment of the novel alloy are studied by observing the microstructure, mechanical properties, and strengthening mechanism. Scanning electron microscopy (SEM) and transmission electron microscopy (TEM) techniques are employed to examine the changes in the microstructure resulting from various solid solution treatments and aging treatments. The best strengthening effect can be achieved when the solubility of the MgSi phase and precipitate β″ (Mg_2_Si phase) is at their maximum. The addition of Er and Zr elements promotes the precipitation of the β″ phase and makes the b″ phase more finely dispersed. The aging strengthening of alloys is a comprehensive effect of the dislocation cutting mechanism and bypass mechanism, the joint effect of diffusion strengthening of Al_3_(Er,Zr) particles and the addition of Er and Zr elements promoting the precipitation strengthening of β″ phases. In this paper, by adding Er and Zr elements and exploring the optimal heat treatment system, the yield strength of the alloy reaches 437 MPa and the tensile strength reaches 453 MPa after solid solution treatment at 565 °C/30 min and aging at 175 °C/10 h.

## 1. Introduction

The 6xxx-series aluminum alloys, mainly composed of Al, Mg and Si, are widely utilized as the principal material in the construction of lightweight automobiles and high-speed trains because they have good formability and corrosion resistance [1]. In addition, the use of aluminum profiles can reduce welding and facilitate transportation. The front and rear bumper crossbeams of car bodies use more extruded aluminum profiles. The mechanical properties of 6xxx-series aluminum alloys are reliant on the effect of several factors such as the composition of the alloy, the extent of solid solubility, and the type of aging treatment applied. With a suitable aging treatment, Al-Mg-Si alloys with Er element addition can be strengthened [2]. The main alloying elements in the 6xxx-series aluminum alloys are Mg and Si, which are combined to form the particles that have a reinforcing effect on the alloys [3]. The precipitation sequence of a typical 6xxx-series aluminum alloy is as follows: supersaturated solid solution (SSS) → atomic clusters → GP region (pre-β″) → β″ → β′ → β [4]. Over the course of the research of recent years, it has been generally accepted that β″ is the reinforcing phase that influences the materials’ ultimate properties. The primary reinforcing elements in the 6xxx-series aluminum alloys are Mg and Si, and the types and numbers of precipitation phases decide the properties of the final formed state and the reinforcing phase [5]. The addition of zinc (Zn) has been found to benefit the artificial age-hardening capability of Al-Mg-Si alloys. Xu et al. [6] found that Zn addition enhanced the aging hardening response ability of Al-Mg-Si alloys in the stage of artificial aging by increasing the formation of precipitates. The strength can be improved if the Zn content is lower than 1.0 wt%. The Mg-Zn atomic clusters formed in the early stage of aging are an important reason for the enhanced response of the alloy. Research has found that the addition of Er to an Al-Mg alloy enhances its strength by more than 20% [7]. By the formation of Al_3_(Er, Zr) particles with a L_12_ structure, Er-Zr composite alloying exhibits synergistic effects [8]. Wu et al. [9] found that Al_3_(Er, Zr) particles coherent with the aluminum matrix can make alloy grain structures stable as well as increase the recrystallization temperature. They can also prevent recrystallization by pinning grains and subgrain boundaries. The addition of the Er element to the alloy can improve its properties without excessively increasing the cost of the alloy material, which makes the high-performance aluminum alloy containing Er have broad application prospects. However, there is limited research on whether Al-Mg-Si-Er-Zr alloys can further improve their strength through solid solution and aging treatment.

To enhance the mechanical properties of Al-Mg-Si alloys and ensure corrosion properties, the heat treatment process of alloys with different compositions has been explored in recent years [10]. High solid solubility can improve strength. The rise in solid solution temperature promotes grain growth as well [11]. High temperatures will increase grain growth irregularity, resulting in various grain sizes and inhomogeneous organization. Inhomogeneous organization influences plates’ mechanical characteristics and surface quality [12,13]. The precipitation of reinforcing phases occurs during the casting and the extrusion process, so it is necessary to carry out the solution treatment and precipitation strengthening by artificial aging so that the alloy achieves the desired properties.

Li et al. [11] investigated the effect of homogenization on the mechanical properties of Al-Mg-Si plates before extrusion. The researchers observed that the tensile strength and elongation of the extruded plates exhibited an increase in response to both the homogenization temperature and the duration of the holding period. When the temperature exceeds a specific critical value, the mechanical properties will experience a significant decline. An insufficient quenching rate after aging can minimize the strength of the alloy. When the rate of quenching decreases, there is a related decline in the activation energy of vacancy diffusion during the aging process, aging kinetics decreases, and the peak hardness after aging decreases [13,14,15]. This study conducted solid solution and aging treatment on hot extruded Al-Mg-Si alloys, and compared their mechanical properties. In addition, the impact of solid solution temperature on the residual phases of Al(FeMn)Si and Mg_2_Si as well as the Al_3_(Er,Zr) precipitation were investigated by scanning electron microscopy (SEM) and transmission electron microscopy (TEM) analysis. By comparing the effects of microstructure and mechanical properties by solid solution aging after hot extrusion on Al-Mg-Si alloys, we can select the optimal solution temperature and peak aging.

## 2. Materials and Methods

### 2.1. Sample Preparation

The chemical composition of the alloy studied in this paper is shown in Table 1. The experimental materials were prepared by alloying pure aluminum with Al-20Si, Al-10Mn, Al-6Er, Al-10Zr (wt%) intermediate alloys, pure Mg, and pure Zn. These metals are melted in a furnace at a temperature of approximately 800 °C. The alloy was homogenized at 300 °C for 10 h, maintained at 555 °C for 20 h, and then extruded into plates of 4 mm thick. The samples were placed in an electrically heated air circulation furnace for the solid solution at 555–585 °C (per 10 °C one regime) for 30 min, quenched rapidly in cold water to produce a supersaturated solid solution, and after that, they were aged at different time intervals in the range of 0–24 h at 165 °C, 175 °C, and 185 °C. Regarding the solid solution treatment, in order to prevent experimental errors caused by powder samples in DSC experiments, an additional 585 °C solid solution temperature was added for further investigation.

### 2.2. Mechanical Property Testing

**Figure 1:**
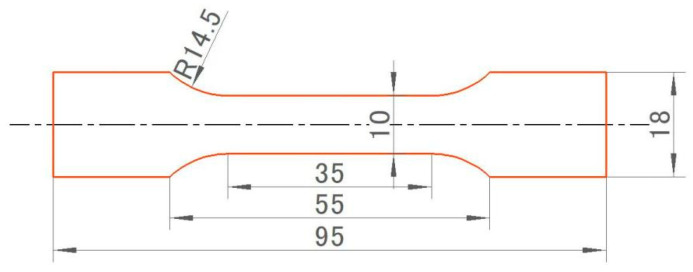
Schematic diagram of room temperature tensile specimen (unit: mm).

Vickers microhardness (HV) tests were carried out using an HXD-1000TM/LCD, (Shanghai, China) hardness tester with a 1 N load applied for 10 s; the results presented here are the average of at least nine measurements. The tensile properties of the specimens were measured in accordance with GB/T 228.1-2021 [16] “Room temperature tensile test methods for metallic materials”, and Figure 1 below depicts the size of the tensile specimens. The fracture morphology was analyzed by an FEI Quanta 650 scanning electron microscope (Eindhoven, North Brabant, The Netherlands) after the tensile test.

### 2.3. Microstructure Characterization

The samples’ microstructures were analyzed via a scanning electron microscope (model FEI Quanta 650, Eindhoven, North Brabant, The Netherlands). The microstructure of the RD-ND surface of the sample was analyzed using an FEI Quanta 650 scanning electron microscope equipped with an EBSD probe. We grinded, polished, and electropolished the samples using 90% (vol%), C_2_H_5_OH, and 10% (vol%) HClO_4_, and immediately washed the sample with sufficient ethanol and air-dried it after electropolishing to avoid secondary corrosion on the surface of the sample. The step size was 0.6 μm. TEM measurements were performed using JEM-2100 F (Tokyo, Japan) with an acceleration voltage of 200 kV for a further microstructural examination. The TEM specimen was mechanically polished to a thickness of approximately 70 microns before being punched into a disc with a diameter of 3 mm. Finally, the sample was twin-jet electropolished in a solution containing 70% methanol and 30% nitric acid (the applied current was set between 80 and 100 mA, and the working temperature was below −30 °C). To avoid data errors, approximately 10 images were used to calculate the size of precipitates.

## 3. Results

### 3.1. Solid Solution

#### 3.1.1. Overheating Temperature

**Figure 2:**
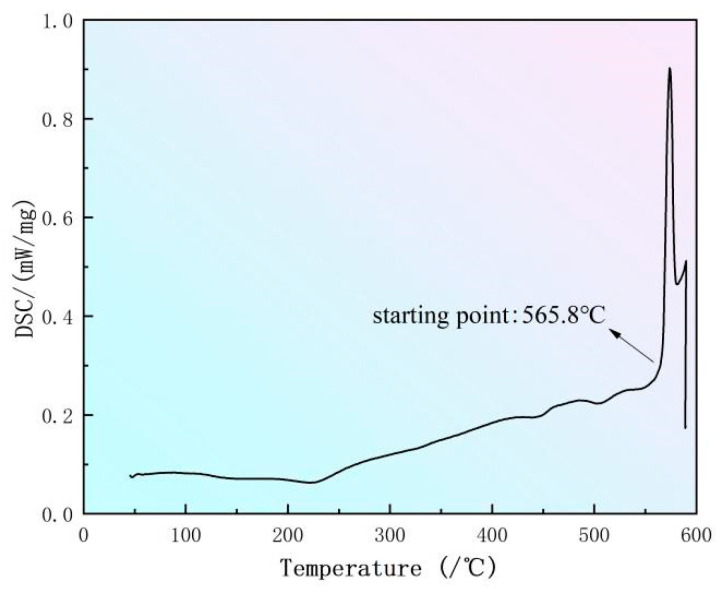
DSC curve of sample.

The result of the DSC analysis of the alloy is shown in Figure 2. The moment it reaches 565.8 °C, the endothermic peak begins, and the low melting point eutectic phases within the alloy begin to melt. It can be inferred that this alloy can be strengthened at a temperature below 565.8 °C. Otherwise, the material may overheat and lose efficacy. To prevent experimental errors caused by powder samples in DSC experiments, the solid solution temperature of 585 °C can be added for further investigation.

#### 3.1.2. Effect of Solid Solution Temperature on Microstructure and Properties of Al-Mg-Si Alloy

**Figure 3:**
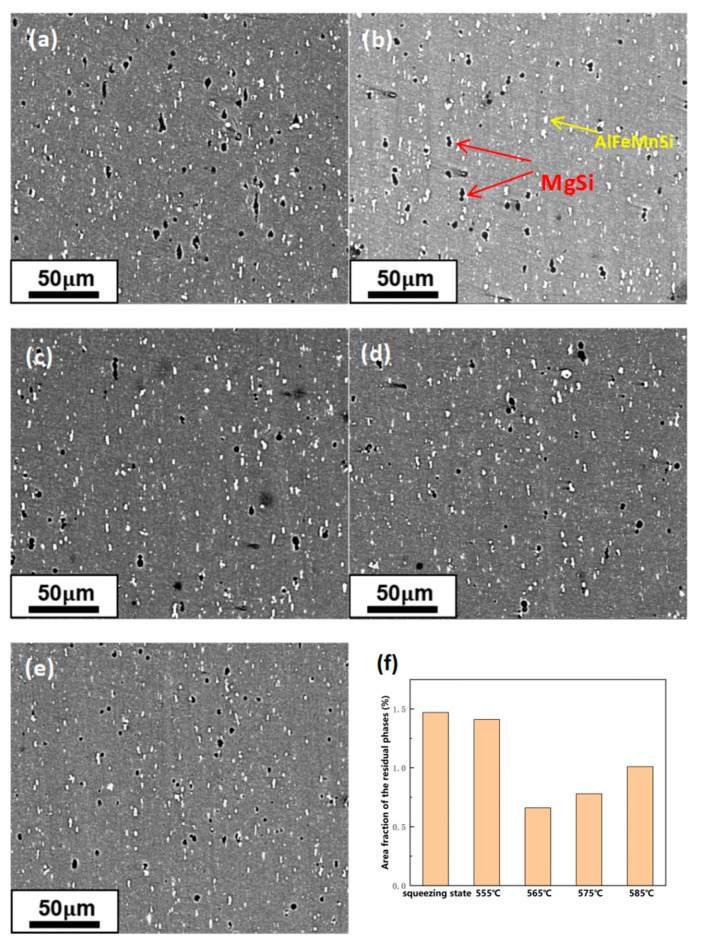
BSE images and residual phases area fraction statistics at different solid solution temperatures: (**a**) T4 extruded state; (**b**) 555 °C; (**c**) 565 °C; (**d**) 575 °C; (**e**) 585 °C; (**f**) Residual phase area fraction statistics.

The BSE images of the specimens in the extruded state and solid solution treated for 30 min at different temperatures are displayed in Figure 3. A significant number of dark MgSi phases and bright AlFeMnSi phases exist in the extruded alloy, which is shown in Figure 3a. The MgSi phases are the main reinforcing phases. By solid solution treatment, MgSi phases can dissolve into the matrix. At this temperature, the AlFeMnSi phases are not easy to dissolve, which affects the alloy’s mechanical properties negatively. These phases are distributed along the extrusion direction, and as a result of the severe deformation caused by the extrusion process, the insoluble phases initially distributed at the grain boundaries are broken into small particles during the hot extrusion process.

Image Pro 6.0 software is used for calculating the fraction of the region occupied by dark residual phases. It can be seen that as the solid solution temperature rises, the total area fraction of residual phases decreases to the lowest at 565 °C, which indicates that the soluble phase in the alloy has dissolved completely. Afterward, the total area fraction of residual phases increases when the temperature reaches 575 °C and 585 °C due to overburning.

#### 3.1.3. Influence of Solid Solution Treatment on Dynamic Recrystallization

**Figure 4:**
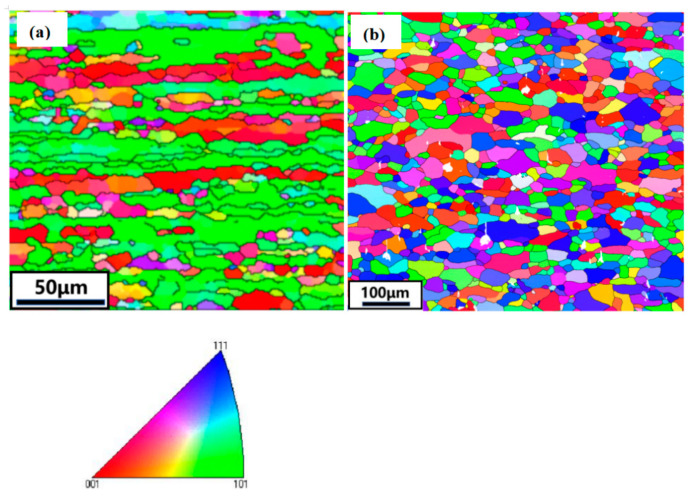
EBSD maps of grain orientation distribution of Al-Mg-Si alloy under (**a**) the extruded state; (**b**) solid solution for 565 °C/30 min.

Figure 4 shows the EBSD analysis of the Al-Mg-Si alloy after extrusion and quenching as well as solution treatment at 565 °C for 30 min. Figure 4a shows that the grains are elongated along the extrusion direction, presenting a typical fibrous shape. The grain orientations are mainly [111] and [101], indicating that there is a clear preferred orientation of the grains, which means there is texture, resulting in an anisotropic performance of the alloy. Figure 4b shows that after solid solution treatment at 565 °C/30 min, the grains change from fibrous to equiaxed, indicating that the degree of recrystallization of the alloy is relatively complete, and the mechanical properties of the alloy are improved.

#### 3.1.4. Influence of Al-Mg-Si Alloy Solid Solution Temperature on Its Mechanical Properties

**Figure 5:**
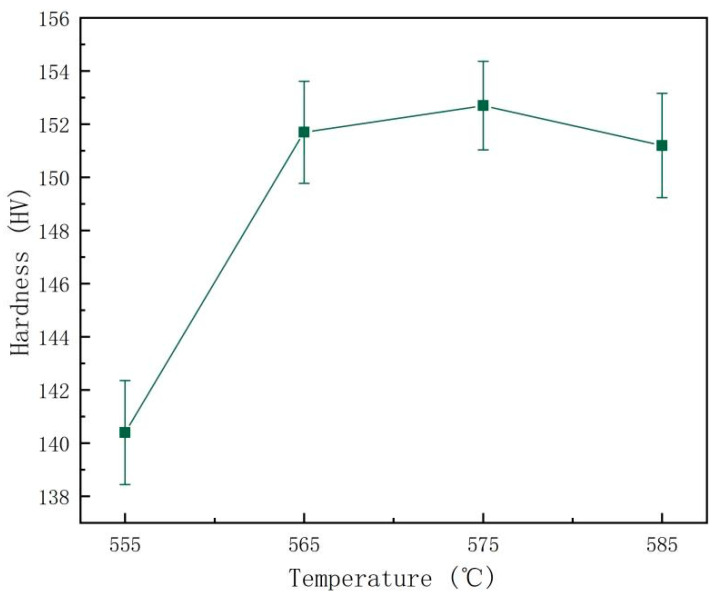
Microhardness curves of the Al-Mg-Si alloy at various solid solution temperatures and aging at 175 °C for 8 h.

Figure 5 depicts the curves of hardness after solid solution treatment at various temperatures. In the temperature range, the specimen’s hardness displays an increasing and then decreasing trend. The specimen’s hardness significantly increases at 565 °C and 575 °C, and continuing to raise the temperature may lead to a decrease in the hardness of the alloy due to overburning.

Tensile tests were carried out on the alloys at different solid solution temperatures and aging at 175 °C for 8 h. The ultimate tensile strength (UTS), yield strength (YS), and elongation (EL) before failure are shown in Table 2. From the results, it can be observed that the mechanical properties of the alloys were optimized at solid solution temperatures of 565 °C and 575 °C. Their UTS and YS were higher than at other temperatures, which corresponded with the hardness curve results. However, the elongation at 575 °C is significantly less than at 565 °C. Considering the overall performance, 565 °C is the optimum solid solution temperature; in this condition, the yield strength reaches 364 MPa, tensile strength reaches 402 MPa, and elongation reaches 13.5.

As the temperature of the solid solution rises, most second phases gradually dissolve back into the matrix, the supersaturation of the matrix gradually increases, and the number of Mg_2_Si strengthened phases precipitated during aging increases. Due to the higher temperature leading to overheating, grain growth increases so that the alloy’s hardness declines. Regarding tensile properties, the solid solution regime was chosen to be 565 °C/0.5 h.

### 3.2. Aging Treatment’s Impact on the Microstructure and Properties of Alloys

**Figure 6:**
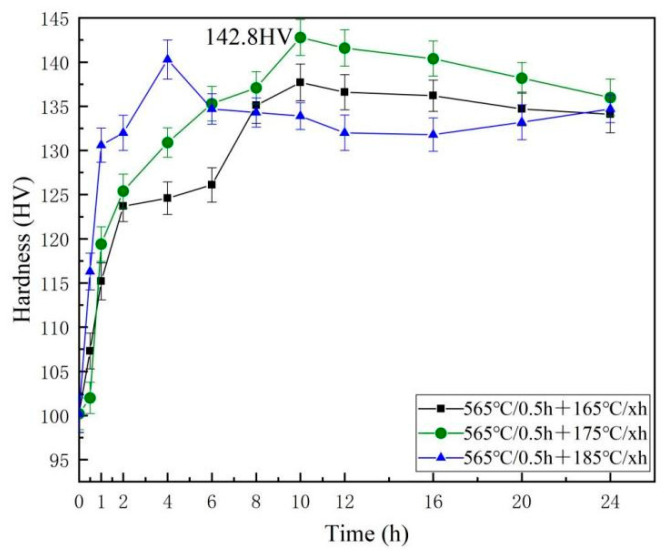
Al-Mg-Si alloy microhardness curves at various aging temperatures.

The hardness graphs after aging at various temperatures are shown in Figure 6. In the early stage of aging, the hardness value of the alloy rapidly increases due to the formation of Mg and Si clusters, which subsequently evolve into GP zones that are coherent with the Al matrix. At this point, the strengthening effect of the alloy will depend on the GP region, and the strength of the alloy will be improved through the shear mechanism of dislocations. As the aging time increases, the GP region transforms into β″ phases, which are the most important strengthening phase; when β″ phases completely precipitate, the maximum strengthening effect is achieved, and the hardness of the alloy is the highest at this time. As the aging time continues to increase, the hardness decreases, which is because β″ precipitates are coarsening. In addition, the results show that, at the same temperature, the hardness of the sample goes up at first, then down as time goes on. With the rise of aging temperature, it will take a shorter time to attain the peak hardness. At 185 °C, which is especially obvious compared with 175 °C, the time required to obtain the aging peak hardness has been reduced from 10 h to 4 h. The hardness raised to 142.8 HV after aging at 175 °C for 10 h, which was higher than any other temperatures. The results of the hardness curves indicate that the best aging condition for Al-Mg-Si alloys in this study is aging at 175 °C for 10 h.

The findings of tensile tests that were conducted on the peak aging state are presented in Table 3. These tests were carried out in order to identify the ultimate tensile strength (UTS), yield strength (YS), and elongation (EL) before fracture. At 175 °C, this alloy obtains the best mechanical properties called the peak aging state. The result is in accordance with the hardness curve results. Their UTS and YS were higher than those of any other temperatures. Compared with the alloy aging directly after extrusion at 175 ± 5 °C × 8 h, the alloy’s UTS, YS, and EL rose by 29.4%, 50.2%, and 17.7%, respectively. This is associated with the precipitation of β″ phases when aging.

**Figure 7:**
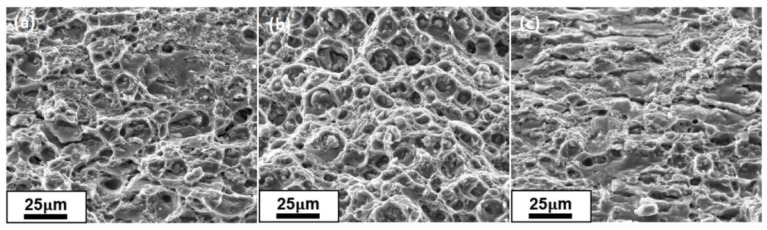
SEM images of the fracture surface morphology of Al-Mg-Si alloy under different aging treatments: (**a**) 175 °C/8 h, (**b**) 175 °C/10 h, (**c**) 175 °C/12 h.

Figure 7 shows the morphology of the tensile fracture in the peak aging state. In all three pictures, there are obvious ligamentous nests, consisting of ligamentous nests and tearing edges, which is a typical example of a toughness fracture. A visible ligamentous nest and tearing edge appear in the fracture of each sample, which is symbolic of toughness fractures. The distribution of ligamentous nests of the sample in the aging state of 175 °C/8 h is not uniform; the ligamentous nests are shallow with tearing edges. In contrast, the ligamentous nests of the peak aging state of 175 °C/10 h have a high density and depth, and there are small ligamentous nests mixed with large ligamentous nests, which have better ductility in this situation. When aging at 175 °C/10 h, the sample’s ligament fossa density is small, with a shallow depth, more tear ribs, and the ductility decreases. The tensile fracture can also be seen at the peak aging state of 175°C/10 h; the comprehensive mechanical properties are better.

**Figure 8:**
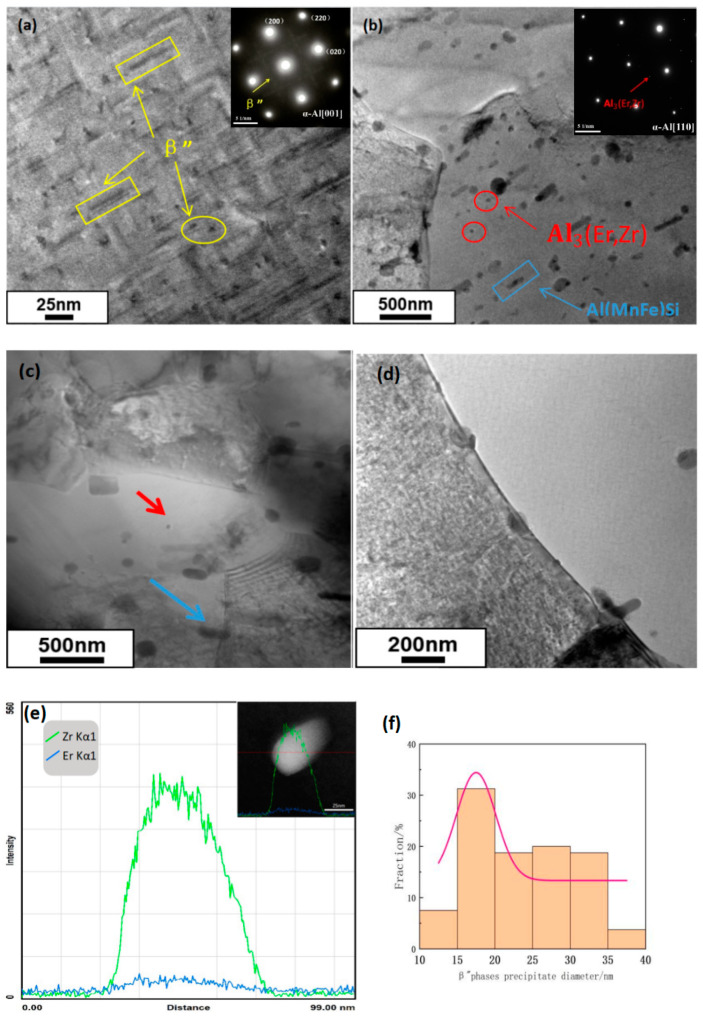
TEM images of T6 alloy: (**a**) β″ precipitate morphology, (**b**) Al_3_(Er,Zr) precipitate morphology, (**c**) bright field; (**d**) grain-boundary precipitates; (**e**) EDS spectra of encircled region in (**d**); (**f**) β″ histogram of phase-length size distribution.

The microstructure of the precipitated phases observed by TEM can explain the evolution of the mechanical properties after aging treatment, and Figure 8a shows the microstructure of the precipitated phases and the diffractograms of selected regions after peak aging at 175 °C/10 h in this Al-Mg-Si alloy. Short bars and grains predominate in the precipitated stages. Two sets of diffraction patterns can be seen in the figure. The prominent diffraction spots are α-Al, and the weak spots are the precipitated phases of Mg_2_Si, indicated by the yellow arrow. For the precipitated phases shown in Figure 8a of multiple TEM photos, the statistical results are shown in Figure 8f; after peak aging treatment, the d of the Mg_2_Si is about 24 nm. As shown in Figure 8b,c, which is the same as in the literature [17,18,19], after aging, Al(MnFe)Si phases with a size of around 200 nm are shaped like short lamellar particles, which are indicated by the blue arrows. There are precipitated phases in fine-grained, granular Al_3_(Er,Zr) phases, as seen in diffractograms of the selected regions with two sets of diffraction patterns. The prominent diffraction spot is α-Al, and the weak spot is the Al_3_(Er,Zr) phase with an L_12_ structure, and the size of the Al_3_(Er, Zr) phase is about 30 nm, indicated by the red arrow. As shown in Figure 8c, the second-phase particles become barriers to restrain dislocations and subgranular borders. By obstructing dislocations and subgranular borders’ movement and stopping them from combining, they delay the start of recrystallization. Odoh et al. [20] suggested that the Mg_2_Si phases in alloys can pin dislocations and negatively affect dislocation motion, increasing the thermally activated energy (Q) and the flow stresses. Zhu et al. [21] suggested that due to higher extrusion resistance, the AA6063 alloy’s flow stress is significantly impacted by additional Mg and Si contents. In this study, the alloy has a high content of Mg and Si, and an excess of large Mg_2_Si particles prevents the movement of dislocations.

In addition, the composition of the grain boundary precipitated phases is shown in Figure 8d. Er, and Zr predominate in the particulate precipitates at the T6 alloy’s grain boundaries. With the addition of Zr and Er elements, the number of grain boundary precipitated phases increases and these are more uniformly distributed. Figure 8e shows the line scanning EDS analysis on the circled phase in Figure 8d, and the results showed that the secondary precipitate phase was rich in Er and Zr, and the signal intensity of Zr was higher than that of Er. This conclusion has also been mentioned in other studies [7].

## 4. Discussion

### 4.1. Influence of Aging Treatment on Precipitation Behavior

The alloy’s mechanical properties depend on the precipitated phases’ size and distribution during aging. In the early aging stage, numerous GP zones can form as nucleation sites for the precipitated phases. GP zones are small and widely dispersed due to the alloy’s significant supersaturation at low temperatures. In this experiment, the optimal solid solution regime of 565 °C/30 min and peak aging of 175 °C/10 h has the best mechanical properties; the UTS, YS, and EL are improved by 29.4%, 50.2%, and 17.4%, respectively. There was no significant improvement in the material’s mechanical properties as further aging occurred. The distribution is more uniform because the quantity of β″ phases in the alloy increases during aging. Because the habitual direction of the β″ phase is <001> Al, there are two characteristic morphologies: needle-like (axial) and spherical (radial) when observing along the <001> Al direction, as indicated by the yellow box and yellow circle markings in Figure 8a. The corresponding selected electron diffraction patterns are shown in the upper right corner illustration. During peak aging, the precipitate β″ phases in the alloy have the highest numerical density and the best reinforcement effect. However, with the purpose of enhancing the alloy’s mechanical properties, it is not useful to age the alloy for an excessively long period of time since the formation of these precipitated phases results in a coarsening phenomenon as the temperature rises.

As can be seen from Figure 8, the peak aging state precipitation β″ phase with short rods and grains uniformly distributed in the matrix, Al_3_(Er, Zr) phases, and Al (MnFe) Si phases diffusely distributed in the matrix can pin dislocations and sub-granular boundaries; because the presence of high-density precipitation hinders the movement of dislocations, higher stress is required during bending.

For heat-treatable strengthened alloys, solid solution and aging treatments are the primary methods for improving the alloy’s mechanical properties, and the precipitation strengthening phases can be maximized by controlling the solid solution temperature and time and aging regime.

### 4.2. Microalloying of Precipitated Er and Zr Phases

The energy held by the matrix is increased when Er and Zr are added to the matrix solid solution. Furthermore, the precipitation phase impedes grain boundary migration, which refines the grain and improves the alloy’s properties [22]. After the solid solution, some intermetallic compounds dissolve in the matrix and create a supersaturated solid solution. During the aging process, large quantities of diffuse phases were produced. As seen in Figure 8d, TEM analysis indicated the appearance of Al_3_(Er, Zr) phases in the alloy, which are dispersed in the aluminum matrix, playing a role in dispersion strengthening. Al_3_(Er, Zr) phases have a small lattice constant and a core-shell structure, which prevents particle coarsening and enhances the mechanical strength and plasticity of the aluminum alloy.

Additionally, these phases work as a matrix for irregular nucleation, encouraging the remaining Al_3_(Er, Zr) and the Mg_2_Si phases to form. In comparison to the matrix alloy, the quantity of the Fe-rich phase reduces as the Er and Zr contents rise, lessening the detrimental effects of grain boundary coarsening and Fe enrichment. Furthermore, the grain boundary precipitation phases can enhance an alloy’s strength by inhibiting the movement of grain boundaries, inhibiting recrystallization, reducing grain size, and producing substructural solid strengthening and precipitation strengthening [23].

The dislocations encounter the precipitated phase particles during their movement, and the dislocation rings may form around the particles or cut through the precipitated phase particles, increasing the dislocation density and enhancing the alloy’s reinforcing effect. TEM analyses in Figure 8c show that there are a large number of dislocations arranged in a disorderly manner around the precipitated phase particles Al_3_(Er, Zr), which leads to dislocation accumulation. Some of these dislocations cut through the precipitated phase particles, while others are bypassed by dislocations. The aging strengthening of this alloy is a comprehensive effect of the cutting mechanism and the bypass mechanism. In addition, the Mg_2_Si phase plays a role in the precipitation strengthening mechanism. The strength of the alloy is enhanced by the synergistic combination of two distinct strengthening methods.

## 5. Conclusions

The effect of solid solution aging treatment on the mechanical properties of Al-Mg-Si alloys and the microalloying impact of Er and Zr on precipitated phases were studied in this paper. Some of the main results are described below.

The alloy possesses the optimum mechanical properties with the following heat treatment regime: solid solution treatment at 565 °C/30 min + aging 175 °C/10 h. Through solid solution aging, recrystallization can be promoted, resulting in the transformation of grains from fibrous to equiaxed after extrusion. Compared with the direct aging immediately after quenching and extrusion, the UTS, YS, and EL are improved by 29.4%, 50.2%, and 17.4%, respectively.

During the process of aging treatment, besides the strengthening effect of the secondary precipitation phase Mg_2_Si, a significant quantity of scattered and co-lattice Al_3_(Er, Zr) phase particles were shown to typically precipitate along grain boundaries and pin dislocations, which can obstruct the movement of dislocation, thereby improving alloy strength.

Through solid solution and aging treatment at 565 °C/30 min + 175 °C/10 h, the Al-Mg-Si alloy was strengthened to 453.0 MPa of tensile strength, 437.0 MPa of yield strength, and 15.5% of elongation by the comprehensive effects of solid solution strengthening, grain refinement, and precipitation strengthening.

## Figures and Tables

**Table 1:** Chemical composition of Al-Mg-Si aluminum alloy plates (wt%).

Alloy	Mg	Si	Fe	Mn	Zn	Zr	Er	Al
Al-Mg-Si alloy	1.1	1.58	0.18	0.59	0.5	0.11	0.1	Bal

**Table 2:** Mechanical properties of the Al-Mg-Si alloy under different solid solution temperatures.

State	Ultimate Tensile Strength, MPa	Yield Strength, MPa	Elongation, %
555 °C/30 min + 175 °C/8 h	374	328	14.0
565 °C/30 min + 175 °C/8 h	402	364	13.5
575 °C/30 min + 175 °C/8 h	408	383	3.5
585 °C/30 min + 175 °C/8 h	360	356	2.5

**Table 3:** Mechanical properties of Al-Mg-Si alloy under peak aging state.

State	Ultimate Tensile Strength, MPa	Yield Strength, MPa	Elongation, %
Direct aging (175 ± 5 °C × 8 h)	350	291	13.20
565 °C/0.5 h +175 °C/8 h	388	382	13.0
565 °C/0.5 h +175 °C/10 h	453	437	15.5
565 °C/0.5 h +175 °C/12 h	428	415	13.5

## Data Availability

Data can be shared online.
